# Identifying Targets for Substance Use Prevention in Young People Exposed to Childhood Adversity: Protocol for a Systematic Review

**DOI:** 10.2196/22368

**Published:** 2020-12-04

**Authors:** Lucinda Rachel Grummitt, Erin Veronica Kelly, Emma Louise Barrett, Katherine M Keyes, Nicola Clare Newton

**Affiliations:** 1 The Matilda Centre for Research in Mental Health and Substance Use The University of Sydney Sydney Australia; 2 Department of Epidemiology Mailman School of Public Health Columbia University New York, NY United States

**Keywords:** adverse childhood experiences, substance use, adolescence, mediation, moderation

## Abstract

**Background:**

Adverse childhood experiences are prevalent robust risk factors for the development of substance use problems. However, less is known about the causal mechanisms that explain these relationships. While directly preventing adverse childhood experiences is ideal, it is not always possible. In such cases, the mechanisms themselves may be amenable to intervention, allowing for the effective prevention of problematic substance use among children exposed to adversity. Identifying such mechanisms is therefore a critical step for efforts aiming to reduce the high individual and societal burdens associated with substance use globally.

**Objective:**

This study aims to systematically identify and synthesize evidence on the modifiable mediators and moderators of the relationship between adverse childhood experiences and substance use outcomes in young people (age 10-24 years).

**Methods:**

A systematic review will be conducted using PubMed, MEDLINE, PsycINFO, Web of Science, and CINAHL databases to determine the modifiable mediators and moderators of the relationship between adverse childhood experiences and substance use in young people. Data from the review will be qualitatively synthesized, unless we identify a sufficient number of studies (at least five) that examine the same type of adversity (eg, physical or sexual abuse) and the same mediator/moderator, in which case a quantitative synthesis (meta-analysis) will be conducted. If a quantitative synthesis is warranted, standardized effect estimates of the indirect (mediated) effect between adverse childhood experiences and substance use outcomes will be combined using a random-effects meta-analysis. Mediators/moderators will be grouped according to a socioecological perspective, using the four levels of individual, interpersonal, community, and public policy/culture.

**Results:**

Electronic searches were completed in August 2019. A total of 4004 studies were included for screening after removing duplicates. After evaluating titles and abstracts against eligibility criteria, a further 3590 studies were excluded, leaving 415 studies for full-text screening. The results of the review are expected to be available by December 2020.

**Conclusions:**

The mechanisms linking adverse childhood experiences and substance use outcomes in young people are vital targets for substance use prevention efforts. This review will provide evidence to inform the development of prevention strategies in order to interrupt the negative life trajectory that can begin with childhood adversity.

**Trial Registration:**

PROSPERO International Prospective Register of Systematic Reviews CRD42020148773; https://www.crd.york.ac.uk/prospero/display_record.php?ID=CRD42020148773

**International Registered Report Identifier (IRRID):**

DERR1-10.2196/22368

## Introduction

Children exposed to adversity are at greater risk of developing substance use problems later in life compared with children not exposed to such adversity [1,2]. Adverse childhood experiences, defined as abuse, neglect, household violence, parental psychopathology, and separation [3], increase the risk of harmful alcohol use by 47% and psychoactive drug use by 64% [1]. The risk is even greater for those exposed to abuse in particular, with meta-analytic estimates demonstrating that these children are twice as likely to develop harmful drinking [4] and have up to two times the odds for any substance abuse [5] compared with those not exposed. Moreover, meta-analytic estimates for individuals exposed to at least four adverse childhood experiences indicate an almost six-fold increase in the odds for illicit drug use and problematic alcohol use and a 10-fold increase in the odds for problematic drug use compared with individuals having no adverse childhood experience exposure [6]. It is therefore no surprise that population attributable risk proportions indicate that elimination of adverse childhood experiences would prevent more than one-quarter of all cases of substance use disorder [7]. Unfortunately, prevalence rates for adverse childhood experiences are alarmingly high, with around 39% of children having experienced an adverse childhood experience globally [7] and high likelihood of experiencing multiple categories of adverse childhood experiences [8,9]. The eradication of adverse childhood experience exposure, while an ultimate goal, may not be readily achievable. Therefore, understanding how best to prevent the negative sequelae resulting from adverse childhood experience exposure is vital in order to lessen the individual, economic [10,11], and global disease burdens [12] attributable to substance use.

Adolescence and emerging adulthood are critical periods for intervention and prevention of substance use problems, given the relatively young age of onset of substance use and development of substance use disorder symptoms [13]. Initiation of substances typically occurs during this period [14] and escalates sharply from early to late adolescence. While trends in adolescent substance use show continuing declines [14,15], there remains a substantial proportion of adolescents who are engaging in harmful substance use. For example, 14% of American 12th graders binge drink fortnightly [15]. This suggests that current prevention approaches may be inadequate for those most at risk for substance use problems. In this respect, young people exposed to adverse childhood experiences represent an important population to target. Adverse childhood experiences substantially increase the odds of early experimentation with substances and early onset of regular use [16,17], which in turn is associated with an increased risk of substance dependence and use disorder [18,19], a more chronic course of dependence [13], and comorbid mental and physical health problems [20]. An estimated 75% of lifetime cases of substance use disorder have their onset before age 24 years [21], highlighting the need to intervene early, prior to the onset of maladaptive patterns of substance use. Given the increased odds of early initiation in those with histories of childhood adversity, intervention prior to young adulthood is especially important.

However, effective prevention of substance use problems in young people with a history of adverse childhood experiences is lacking. This may be in part due to a lack of clarity around specific targets for prevention in this population. Mechanisms that mediate or moderate the relationship between adverse childhood experiences and substance use outcomes and are amenable to change reflect key targets for prevention. Existing research points to such mechanisms at the individual, interpersonal, and community levels of behavior. Specifically, early adversity has been linked to changes in inhibitory control and reward processing, which in turn predict vulnerability to substance use disorder [22]. Differences in executive control were indeed found to mediate the relationship between adverse childhood experiences and substance use in adolescents [23]. Internalizing and externalizing symptoms appear to also be involved in the risk for substance use problems associated with child adversity [24]. Additionally, there is some evidence that emotion regulation processes mediate the link between emotional abuse and substance use in adults [25]. Interpersonal factors, such as social support [26] and relationships with parents/caregivers [27,28] and peers [28], have also been implicated as mechanisms in the relationship between childhood adversity and substance use. At the community level, preliminary evidence indicates that a sense of school belonging may moderate the effect of adverse childhood experiences on adolescent cigarette smoking, with a higher sense of school belonging protecting against substance use [29].

The literature reviewed above provides some candidates for intervention in the pathway from childhood adversity to substance use outcomes. However, the existing literature on mechanisms involved in the relationship between adverse childhood experiences and substance use outcomes is limited in three important ways. First, studies often examine a single mediator or moderator, or a single type of adversity, despite evidence of multiple mechanisms that contribute to the relationship [26,30] and evidence that children experiencing one type of adverse childhood experience are often exposed to multiple categories [9]. To our knowledge, no systematic effort to identify the range of mediators/moderators in the relationship between adverse childhood experiences and substance use outcomes has been undertaken, hindering our ability to understand the potentially broad range of factors involved in this pathway. Second, longitudinal studies delineating the mechanisms linking adverse childhood experiences and substance use problems are rare. Reliance on cross-sectional data requires substantial and often untenable assumptions to establish a developmental relationship between exposure and substance use outcomes. Longitudinal data upholds the assumptions of mediation analysis, namely, that the temporal relationship between variables is correct [31]. Third, many studies examining the mechanisms that operate between adverse childhood experiences and outcomes focus on static factors that are not amenable to change via traditional strategies, such as genes [32], race/ethnicity [33,34], sex [35], neurobiological mechanisms [36], and brain structure/function [37]. While such research aids our understanding, identifying targets amenable to intervention is a critical next step in preventing the negative sequelae associated with adverse childhood experiences.

This study aims to address these gaps by systematically identifying and synthesizing evidence on the modifiable mediators and moderators of the relationship between adverse childhood experiences and substance use outcomes in young people. Specifically, through a systematic review of the literature, this study aims to determine what modifiable factors mediate or moderate the relationship between childhood adversity and substance use outcomes in young people (age 10-24 years).

## Methods

### Registration

This protocol adheres to the Preferred Reporting Items for Systematic Review and Meta-Analysis Protocols (PRISMA-P) 2015 statement [38]. The protocol has been registered in the PROSPERO registry (University of York, registration number: CRD42020148773).

### Eligibility Criteria

For this review, eligibility criteria are defined using population, intervention/exposure, comparator, outcome, and study characteristics (PICOS) [39]. The inclusion criteria are defined in Textbox 1 [40-45].

**Eligibility criteria for studies to be included in the systematic review and synthesis.**

**Population**

Included studies must include human participants (no animal studies). Participants must have experienced childhood adversity between the ages of 0 and 18 years, and must have a measured substance use outcome between the ages of 10 and 24 years, corresponding to an inclusive definition of adolescence [40] and aligning with the World Health Organization’s definition of “young person” [41].

**Intervention/Exposure**

Studies must include a measure of childhood adversity, occurring between the ages of 0 and 18 years. Childhood adversity is defined here as experiences measured by the CDC-Kaiser Permanente Adverse Childhood Experiences Study [3] as follows: from age 0 to 18 years, emotional, physical, or sexual abuse; emotional or physical neglect; mother treated violently; a member of the household engaged in substance abuse, experienced mental illness, or went to prison; and parents separated or divorced. Moreover, it involves three items proposed by Finkelhor et al as follows: being a victim of bullying, experiencing social isolation/rejection, and prolonged loneliness [42]. These additional items have been included owing to evidence suggesting that they increase the prediction of mental health outcomes when added to the adverse childhood experience survey items [42]. For the purposes of this study, parental psychopathology (substance abuse and mental illness) must have occurred during the child’s lifetime between the ages of 0 and 18 years.

**Comparator**

No comparator/control group required for inclusion.

**Outcomes**

Studies must include a substance use outcome between the ages of 10 and 24 years. This includes alcohol, tobacco, psychoactive drugs, and nonmedical use of prescription drugs and any of the following:

- Initiation and age of initiation of substance use.
- Frequency of substance use.
- Problem substance use or abuse, defined as the presence of any of the following: failure to fulfil major obligations at work, school, or home; recurrent use in situations in which it is physically hazardous; recurrent substance-related legal problems; and continued use despite persistent social or interpersonal problems.
- Quantity of substance use, including single occasion risky drinking (binge drinking/heavy episodic drinking, defined here as at least four standard drinks on any one occasion).
- Substance use disorder/dependence. The definition for this outcome will be according to the diagnostic criteria set out in the Diagnostic and Statistical Manual of Mental Disorders (DSM) version that was in use at the time the study outcome data were collected (either DSM-IV [43] or DSM V [44]).

**Study Characteristics**

Studies must include a mediation/moderation analysis of at least one factor that is modifiable after birth. We consider a mediation analysis to be present if the authors test the indirect effect from the adverse childhood experience to the substance use outcome via a hypothesized mediator. In mediation analyses, we do not require studies to first demonstrate a significant direct effect from the adverse childhood experience to the substance use outcome. This is in recognition of consensus among mediation researchers that if the direct effect is presumed small or temporally distant to the outcome, it need not be significant for mediation to be established [45]. This is plausible in the case of childhood adversity and substance use, whereby the risk conferred from distal factors, such as adverse childhood experiences, may operate through more proximal risk factors that occur in adolescence, thus decreasing the magnitude of the association between childhood adversity and substance use outcomes. We consider a moderation analysis to be present if authors test the interaction between the adverse childhood experience and the proposed moderator.

In addition, studies must be published in English, must be peer-reviewed, must employ a longitudinal study design, and must be original research. Full-text studies must be published from January 1, 1998, to August 14, 2019. The year 1998 was chosen as the historical cut-off point to include only studies published following the CDC Kaiser Permanente Adverse Childhood Experiences study [3].

Specific exclusion criteria were identified. Studies will be excluded if they meet any of the following conditions: evaluation of intervention or treatment outcomes only; assessment of scale formation only; reporting of the incidence of substance use only; and review of the literature. In addition, studies that presume physical neglect owing to poverty status or another income-related measure will be excluded if they do not examine another adverse childhood experience. This was due to the understanding that a family’s income does not necessarily reflect whether a child’s basic physical needs of food, shelter, adequate medical care, and clothing are met.

### Information Sources

Electronic searches will be conducted in PubMed, MEDLINE, PsycINFO, Web of Science, and CINAHL from 1998 to August 2019. Two databases (MEDLINE and PubMed) will be searched without English language restriction to determine whether relevant studies published in other languages are being excluded. Searches will be rerun in 2020 prior to data analysis to identify any relevant studies published since the initial searches were conducted. Table 1 provides the search terms used for MEDLINE, which will be replicated for other databases. Database-specific Medical Subject Heading (MeSH) searches will be generated where exact matches are unavailable. Full search terms for each database are presented in Multimedia Appendix 1.

**Table 1:** MEDLINE search strategy.

Number	Term
1	life change events/
2	adverse childhood experiences/ or domestic violence/ or exp child abuse/ or physical abuse/
3	((childhood or adolescent) adj3 advers*).tw.
4	(child* or life or early) adj2 stress.tw.
5	bullying/ or cyberbullying/
6	social isolation/
7	((family or parent*) adj3 (substance or alcohol* or drug or smok* or depression or illness or suicid* or jail or prison)).tw.
8	divorce/ or family conflict/ or family separation/
9	(trauma* or maltreat* or assault* or violen* or molest* or neglect* or victim* or isolat* or reject* or mistreat* or poverty or depriv* or abus* or lonel*).tw.
10	1 or 2 or 3 or 4 or 5 or 6 or 7 or 8 or 9
11	resilience, psychological/
12	adaptation, psychological/
13	(adapt* or protect* or resilien* or mediat* or moderat*).tw.
14	protective factors/
15	11 or 12 or 13 or 14
16	substance-related disorders/ or exp alcohol-related disorders/ or alcoholic intoxication/ or alcoholism/ or binge drinking/ or amphetamine-related disorders/ or cocaine-related disorders/ or drug overdose/ or inhalant abuse/ or marijuana abuse/ or exp opioid-related disorders/ or phencyclidine abuse/ or substance abuse, intravenous/ or substance abuse, oral/ or “tobacco use disorder”/
17	((substance or alcohol* or tobacco or drug or smok*) adj3 (misuse* or initiat* or abus* or problem or heavy or binge or disorder* or dependen* or frequen*)).ti,ab.
18	16 or 17
19	cohort studies/ or longitudinal studies/ or follow-up studies/ or prospective studies/ or retrospective studies/ or cohort.ti,ab. or longitudinal.ti,ab. or prospective.ti,ab. or retrospective.ti,ab.
20	10 and 15 and 18 and 19
21	child* or adolescen* or teen* or youth* or pediatr* or paediatr* or young or emerging or youth).tw
22	20 and 21
23	limit 22 to ((“all child (0 to 18 years)” or “young adult (19 to 24 years)”) and English)
24	limit 23 to yr=“1998 -current”

If additional information is required from authors of studies identified by the review, the corresponding author of that study will be contacted by email to provide this information. After 3 weeks from the date of the first contact and one follow-up email, if no response has been received from the authors, we will deem them unreachable and proceed with our analysis. If any of the inclusion criteria cannot be confirmed, the study will be excluded.

### Study Records

#### Data Management

Studies identified in the databases will be exported to Covidence [46] and duplicates will be removed by the software.

#### Selection Process

Researcher 1 will screen 100% of the titles and abstracts for inclusion in the review. A second and third researcher will screen 5% of the titles and abstracts, and proportionate agreement will be calculated through the systematic review software [46]. This represents the number of votes in agreement divided by the total number of votes. If discrepancies exist between scores, they will be resolved through consultation between the three researchers. If proportionate agreement is less than 90%, the three reviewers will review the screening process and resolve any ambiguities that may be causing the discrepancies. A second researcher will screen additional studies until agreement is above 90%.

Full-text studies will be obtained for studies deemed eligible for inclusion. Two researchers will read all full-text studies. Discrepancies in the scores of the two researchers will be resolved through consultation, and if required, a third researcher will be included.

#### Data Collection Process and Data Items

From the studies included in the final selection, researcher 1 will independently extract author information, publication year, study characteristics (sample size, age of participants, gender, length of follow-up, and location), substance use outcomes (age of initiation, any use, frequency of use, problem use, heavy use or binge drinking, abuse, and disorder/dependence), characteristics of child adversity (type, age of exposure, and duration of exposure where available), mediators examined, and moderators examined. A summary of the findings for each mediator/moderator will be extracted, including statistical significance and the effect size of the mediated and/or moderated effect.

### Risk of Bias in Individual Studies

Study quality will be assessed using the Joanna Briggs Institute Critical Appraisal Checklist for Studies Reporting Prevalence Data [47]. The checklist has nine questions that assess six domains. One point is allocated per question, and points are combined as a measure of overall study quality, allowing comparison across studies. This critical appraisal tool was chosen as it is well suited to the type of studies that will be extracted from the review, that is, as the review is focused on the presence of mediating factors between an exposure and outcome and not involved in evaluating treatments or interventions, this tool is useful because it does not include questions assessing the appropriateness of randomization procedures, blinding, and intervention integrity that are found in other tools assessing quantitative study quality. Two reviewers will complete the critical appraisal for all studies. Inconsistencies will be resolved through consultation between the two reviewers. The risk of bias of studies will be reported in a narrative synthesis.

### Data Synthesis

We will synthesize mediators and moderators. These will be grouped according to the socioecological model [48], at the individual, interpersonal, organizational/community, and public policy levels. If there is a sufficient number of studies (at least five) that examine the same category of adversity and the same mediator/moderator, a quantitative synthesis will be conducted. If a quantitative review is warranted, standardized effect estimates of the indirect (mediated) effect between the adverse childhood experience and the substance use outcome will be combined using a random-effects meta-analysis. A random-effects approach was chosen as given the likely heterogeneity in samples based on the broad inclusion criteria of the review, it is likely there will be a range of different effect sizes across studies that are not simply due to sampling error. If conditions are not met for a quantitative review, a narrative synthesis will be conducted.

### Confidence in Cumulative Evidence

The GRADE (Grading of Recommendations, Assessment, Development, and Evaluations) approach will be used to assess the strength of the evidence overall [49].

### Ethical Approval

This study does not include human or animal participants and thus does not require approval from ethical review boards.

## Results

Electronic searches were conducted in August 2019. After removing duplicates, 4005 studies were included for screening. On evaluating titles and abstracts against eligibility criteria, a further 3590 studies were excluded, leaving 415 studies for full-text screening. The results of the review are expected to be available by December 2020 (Table 2).

**Table 2:** Progress and timeline (adapted from the PROSPERO International Prospective Register of Systematic Reviews).

Month, Year	Aug-Dec, 2019	Jan-Mar, 2020	Apr-Jun, 2020	Jul-Sep, 2020	Oct-Dec, 2020
Preliminary searches	✓				
Piloting of the study selection process	✓				
Formal screening of search results against eligibility criteria		✓	✓	✓	
Data extraction				✓	
Risk of bias assessment					✓
Data analysis					✓

## Discussion

Substance use and mental disorders are the leading causes of disease burden in young people and are associated with 3.4 million years of health lost [50]. In order to reduce this high burden, prevention must be tailored toward those most at risk for problematic substance use. In this respect, young people with histories of adverse childhood experiences are an important population to target, given the prevalence of adverse childhood experiences and the strong relationship these experiences have with increased risk for substance use. By examining and synthesizing evidence regarding the mechanisms that underlie this relationship, this review will provide valuable knowledge to inform the development of prevention programs to be delivered to this population. Beyond prevention, knowledge of mechanisms in the relationship between adverse childhood experiences and substance use outcomes in young people has important implications for clinical assessment, case formulation, and treatment approaches. Early intervention and treatment may benefit from addressing the mechanisms identified in this review. Moreover, these mediators/moderators may impact treatment success, potentially shedding light on variability in treatment outcomes and offering new opportunities to increase effectiveness. This is especially important as there are minimal evidence-based integrated treatment options for young people with co-occurring traumatic stress and substance use. This review thus seeks to improve outcomes for young people exposed to adverse childhood experiences and interrupt the negative life trajectory that can start with childhood adversity.

## Appendix

Multimedia Appendix 1 Database search terms.
