# Supplementary material for: Identifying Targets for Substance Use Prevention in Young People Exposed to Childhood Adversity: Protocol for a Systematic Review
Source: JMIR Res Protoc. 2020 Dec 4;9(12):e22368. doi: 10.2196/22368 (PMC7748964; doi:10.2196/22368)
Supplement: Multimedia Appendix 1 [file resprot_v9i12e22368_app1.pdf]

Supplementary file: Database search terms

Database: Medline

Childhood Adversity	1	Life Change Events/
	2	adverse childhood experiences/ or domestic violence/ or exp child abuse/ or physical abuse/
	3	((childhood or adolescent) adj3 advers*).tw.
	4	(child* or life or early) adj2 stress.tw.
	5	bullying/ or cyberbullying/
	6	Social Isolation/
	7	((Family or parent*) adj3 (substance or alcohol* or drug or smok* or depression or illness or suicid* or jail or prison)).tw.
	8	divorce/ or family conflict/ or family separation/
	9	(trauma* or maltreat* or assault* or violen* or molest* or neglect* or victim* or isolat* or reject* or mistreat* or poverty or depriv* or abus* or lonel*).tw.
	10	1 or 2 or 3 or 4 or 5 or 6 or 7 or 8 or 9
Mediator/ moderator	11	Resilience, Psychological/
	12	adaptation, psychological/
	13	(adapt* or protect* or resilien* or mediat* or moderat*).tw.
	14	protective factors/
	15	11 or 12 or 13 or 14
Substance use outcomes	16	substance-related disorders/ or exp alcohol-related disorders/ or alcoholic intoxication/ or alcoholism/ or binge drinking/ or amphetamine-related disorders/ or cocaine-related disorders/ or drug overdose/ or inhalant abuse/ or marijuana abuse/ or exp opioid-related disorders/ or phencyclidine abuse/ or substance abuse, intravenous/ or substance abuse, oral/ or "tobacco use disorder"/
	17	((substance or alcohol* or tobacco or drug or smok*) adj3 (misuse* or initiat* or abus* or problem or heavy or binge or disorder* or dependen* or frequen*)).ti,ab.
	18	16 or 17
Study design	19	cohort studies/ or longitudinal studies/ or follow-up studies/ or prospective studies/ or retrospective studies/ or cohort.ti,ab. or longitudinal.ti,ab. or prospective.ti,ab. or retrospective.ti,ab.
	20	10 and 15 and 18 and 19
Age range	21	Child* or adolescen* or teen* or youth* or pediatr* or paediatr* or young or emerging or youth).tw
	22	20 and 21
	23	limit 22 to (("all child (0 to 18 years)" or "young adult (19 to 24 years)") and english)
	24	limit 23 to yr="1998 -Current"

Database: PsycINFO

Childhood Adversity	1	exp childhood adversity/
	2	((childhood or adolescent) adj3 advers*).tw.
	3	((child* or life or early) adj2 stress).tw.
	4	bullying/ or cyberbullying/
	5	social isolation/
	6	((Family or parent*) adj3 (substance or alcohol* or drug or smok* or depression or illness or suicid* or jail or prison)).tw.
	7	divorce/ or marital separation/ or life changes/
	8	exp family conflict/
	9	(trauma* or maltreat* or assault* or violen* or molest* or neglect* or victim* or isolat* or reject* or mistreat* or poverty or depriv* or abus* or lonel*).tw.
	10	child abuse/ or abandonment/ or child neglect/ or child welfare/ or domestic violence/ or emotional abuse/ or physical abuse/ or sexual abuse/
	11	1 or 2 or 3 or 4 or 5 or 6 or 7 or 8 or 9 or 10
Mediator/ moderator	12	"resilience (psychological)"/
	13	exp social adjustment/
	14	(adapt* or protect* or resilien* or mediat* or moderat*).tw.
	15	protective factors/
	16	adaptive behaviour/
	17	12 or 13 or 14 or 15 or 16
Substance use outcomes	18	exp drug abuse/ or addiction/ or drug addiction/ or drug overdoses/ or intravenous drug usage/ or prescription drug misuse/
	19	exp "substance use disorder"/
	20	exp Alcoholism/ or exp Alcohol Abuse/ or exp Drug Dependency/
	21	binge drinking/ or alcohol intoxication/
	22	((substance or alcohol* or tobacco or drug or smok*) adj3 (misuse* or initiat* or abus* or problem or heavy or binge or disorder* or dependen* or frequen*)).ti,ab.
	23	exp marijuana usage/ or "cannabis use disorder"/
	24	cocaine/
	25	exp amphetamine/
	26	exp opiates/ or "opiod use disorder"/
	27	exp "Tobacco Use Disorder"/
	28	18 or 19 or 20 or 21 or 22 or 23 or 24 or 25 or 26 or 27
Study design	29	cohort studies/ or longitudinal studies/ or follow-up studies/ or prospective studies/ or retrospective studies/ or cohort.ti,ab. or longitudinal.ti,ab. or prospective.ti,ab. or retrospective.ti,ab.
Age range	30	(Child* or adolescen* or teen* or youth* or pediatr* or paediatr* or young or emerging or youth).tw.
	31	11 and 17 and 28 and 29 and 30
	32	limit 31 to (english and human)

Database: PubMed

Child adversity	1	((((((((((((life change events[MeSH Terms]) OR adverse childhood experiences[MeSH Terms]) OR domestic violence[MeSH Terms]) OR physical abuse[MeSH Terms]) OR bullying[MeSH Terms]) OR social isolation[MeSH Terms]) OR divorce[MeSH Terms]) OR family conflict[MeSH Terms]) OR family separation[MeSH Terms]) OR child* advers*[Text Word]) OR adolescen* advers*[Text Word]) OR ((family[Text Word] OR parent*)[Text Word] AND (substance[Text Word] OR alcohol*[Text Word] OR drug[Text Word] OR smok*[Text Word] OR depressi*[Text Word] OR illness[Text Word] OR suicid*[Text Word] OR jail[Text Word] OR prison)[Text Word])) OR (trauma*[Text Word] OR maltreat*[Text Word] OR assault*[Text Word] OR violen*[Text Word] OR molest*[Text Word] OR neglect*[Text Word] OR victim*[Text Word] OR isolat*[Text Word] OR reject*[Text Word] OR mistreat*[Text Word] OR poverty[Text Word] OR depriv*[Text Word] OR abus*[Text Word] OR lonel*[Text Word])
Mediators	2	(((("Resilience, Psychological"[Mesh]) OR "Adaptation, Psychological"[Mesh]) OR "Protective Factors"[Mesh])) OR ((adapt*[Text Word] OR protect*[Text Word] OR resilien*[Text Word] OR mediat*[Text Word] OR moderat*[Text Word]))
Substance use outcomes	3	((((substance[Title/Abstract] OR alcohol*[Title/Abstract] OR tobacco[Title/Abstract] OR drug[Title/Abstract] OR smok*)[Title/Abstract] AND (misuse*[Title/Abstract] OR initiat*[Title/Abstract] OR abus*[Title/Abstract] OR problem[Title/Abstract] OR heavy[Title/Abstract] OR binge[Title/Abstract] OR disorder*[Title/Abstract] OR dependen*[Title/Abstract] OR frequen*))[Title/Abstract])) OR (("Substance-Related Disorders"[Mesh]) OR "Binge Drinking"[Mesh])
Study type	4	((cohort[Title/Abstract] OR longitudinal[Title/Abstract] OR prospective[Title/Abstract] OR retrospective[Title/Abstract])) OR ((((("Cohort Studies"[Mesh]) OR "Follow-Up Studies"[Mesh]) OR "Prospective Studies"[Mesh]) OR "Longitudinal Studies"[Mesh]) OR "Retrospective Studies"[Mesh])
Age range	5	(Child*[Text Word] OR adolescen*[Text Word] OR teen*[Text Word] OR youth*[Text Word] OR pediatr*[Text Word] OR paediatr*[Text Word] OR young[Text Word] OR emerging[Text Word] OR youth[Text Word])
	6	1 and 2 and 3 and 4 and 5
	7	Filters activated: Humans, English, Child: birth-18 years, Young Adult: 19-24 years.

Database: Web of Science

	9	(#7 AND #6 AND #5 AND #4 AND #3) AND LANGUAGE: (English)
		Indexes=SCI-EXPANDED, SSCI, A&HCI, CPCI-S, CPCI-SSH, ESCI, CCR-EXPANDED, IC Timespan=All years
	8	#7 AND #6 AND #5 AND #4 AND #3
Age range	7	ALL=(child* OR adolescen* OR teen* OR youth* OR pediatr* OR paedi atr* OR young OR emerging OR youth)
Study type	6	ALL=("cohort studies" OR "longitudinal studies" OR "follow-up studies" OR "prospective studies" OR "retrospective studies" OR cohort OR longitudinal OR prospective OR retrospective)
Substance use outcomes	5	ALL=((("substance-related disorders" OR "alcohol-related disorders" OR "alcoholic intoxication" OR "alcoholism" OR "amphetamine-related disorders" OR "cocaine-related disorders" OR "binge drinking" OR "drug overdose" OR "inhalant abuse" OR "marijuana abuse" OR "opioid-related disorders" OR "substance abuse" OR "tobacco use disorder") OR ((substance OR alcohol* OR tobacco OR drug OR smok*) "NEAR" (misuse* OR initiat* OR abus* OR problem OR heavy OR binge OR disorder* OR dependen* OR frequen*))))
Mediator/ moderator	4	ALL=(resilien*or adapt* or protect* or mediat* or moderat*)
	3	#2 OR #1
Child adversity	2	ALL=(((child* OR adolescen*) "NEAR" advers*) OR ((child* OR life OR early) "NEAR" stress) OR ((family OR parent*) "NEAR" (substance OR alcohol* OR drug OR smok* OR depressi* OR illness OR suicid* OR jail OR prison) OR (trauma* OR maltreat* OR assault* OR violen* OR molest* OR neglect* OR victim* OR isolat* OR reject* OR mistreat* OR poverty OR depriv* OR abus* OR lonel*)))
	1	ALL=("life change events" OR "adverse childhood experiences" OR "domestic violence" OR "child abuse" OR "physical abuse" OR "sexual abuse" OR bullying OR cyberbullying OR "social isolation" OR divorce OR "family conflict" OR "family separation")

Database: CINAHL

	S23	S10 AND S14 AND S18 AND S21 AND S22
Age ranges	S22	Child* or adolescen* or teen* or youth* or pediatr* or paediatr* or young or emerging or youth
	S21	S19 OR S20
Study type	S20	cohort or longitudinal or prospective or retrospective
	S19	(MH "Prospective Studies+")
	S18	S15 OR S16 OR S17
Substance use outcomes	S17	((substance or alcohol* or tobacco or drug or smok*) N3 (misuse* or initiat* or abus* or problem or heavy or binge or disorder* or dependen* or frequen*))
	S16	(MH "Overdose")
	S15	(MH "Substance Use Disorders+")
	S14	S11 OR S12 OR S13
Mediator/ moderator	S13	adapt* or protect* or resilien* or mediat* or moderat*
	S12	(MH "Adaptation, Psychological")
	S11	(MH "Hardiness")
	S10	S1 OR S2 OR S3 OR S4 OR S5 OR S6 OR S7 OR S8 OR S9
Child adversity	S9	(trauma* or maltreat* or assault* or violen* or molest* or neglect* or victim* or isolat* or reject* or mistreat* or poverty or depriv* or abus* or lonel*)
	S8	((Family or parent*) N3 (substance or alcohol* or drug or smok* or depression or illness or suicid* or jail or prison))
	S7	((child* or adolescen*) N3 advers*)
	S6	((child* or life or early) N2 stress)
	S5	(MH "Family Conflict")
	S4	(MH "Social Isolation+")
	S3	(MH "Bullying+") OR (MH "Cyberbullying")
	S2	(MH "Life Change Events") OR (MH "Death+") OR (MH "Divorce")
	S1	(MH "Adverse Childhood Experiences") OR (MH "Domestic Violence+") OR (MH "Exposure to Violence") OR (MH "Child Abuse+") OR (MH "Divorce") OR (MH "Poverty")
		Limiters - English Language; Age Groups: Adult: 19-44 years, All Child
